# Unraveling the coordination isomerism by ligand hyperfine NMR shifts

**DOI:** 10.1039/d5sc09905f

**Published:** 2026-03-04

**Authors:** Dora Cidlinská, Jan Chyba, Markéta Munzarová, Yevgen Yurenko, Jan Novotný, Radek Marek

**Affiliations:** a CEITEC-Central European Institute of Technology, Masaryk University Kamenice 5 CZ-62500 Brno Czechia jan.novotny@ceitec.muni.cz radek.marek@ceitec.muni.cz; b Department of Chemistry, Faculty of Science, Masaryk University Kamenice 5 CZ-62500 Brno Czechia; c National Center for Biomolecular Research, Faculty of Science, Masaryk University Kamenice 5 CZ-62500 Brno Czechia

## Abstract

The hyperfine (Curie) NMR shifts of ligand atoms in open-shell coordination compounds report subtle details of the spin distribution around the central metal atom. In this work, we propose hyperfine NMR shifts as simple and extremely sensitive indicators of the ligand coordination geometry. This is demonstrated for equatorial *versus* axial isomers of neutral octahedral [Ru(*acac*)Cl_2_L_2_] compounds, rationalized by two distinct mechanisms of transmission of the spin density unraveled using density-functional theory analysis. The positional interchange of the two chlorides and the two pnictogen-based ligands (L) induces modifications in the singly occupied molecular orbital composition and the related Fermi-contact hyperfine interactions of the probed atoms of the *acac* ligand, resulting in distinct ^1^H and ^13^C NMR spectral fingerprints. The demonstrated symmetry-driven spin-transmission mechanisms have general validity, which offers hyperfine NMR shifts as a tool to probe the geometry of various classes of coordination compounds, including transition-metal catalysts and metalloenzymes.

## Abstract 2

Paramagnetic NMR reveals how ligand positioning in transition-metal complexes governs SOMO symmetry and spin-delocalization pathways, leading to distinct Fermi-contact NMR shifts.

## Graphical abstract

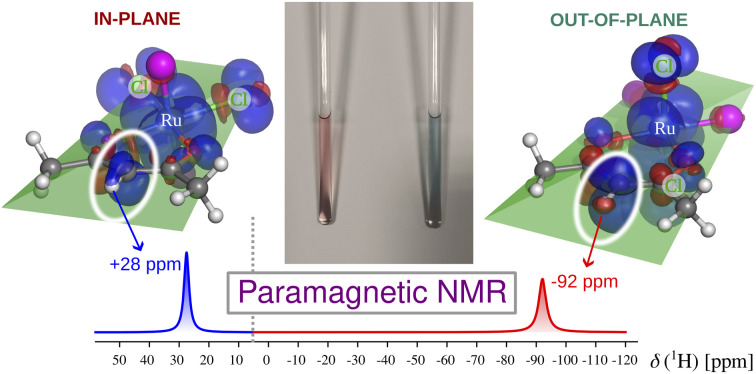

## 1 Introduction

Transition-metal coordination compounds are of immense importance for the quality of our lives and modern technologies. In addition to being parts of natural metalloenzymes,^1,2^ they are used in medicine as anticancer metallodrugs^3,4^ and magnetic resonance contrast agents.^5^ They also represent many essential components of catalytic systems in technologies for the conversion of matter^6^ and energy harvesting.^7,8^

Their applications require syntheses with control over the coordination geometry of the ligands and related structural characterization studies. The most traditional technique used to determine the ligand arrangement around the metal atom is X-ray diffraction (XRD).^9^ However, this technique suffers from the need to prepare single crystals, as powder XRD is still far from routine.^10,11^ For open-shell systems, Mössbauer spectroscopy^12^ is a very powerful tool for determining the local coordination geometry and the oxidation state of the metal. However, this technique has its own limitations for general use. The most easy-to-apply and inexpensive technique is UV-Vis spectroscopy. UV-Vis spectra reflect different electronic excitations between occupied and vacant orbitals for different isomers and thus report the electronic arrangement provided by the coordination geometry.^13,14^ However, experimental interpretation of the bands in the visible spectral range typically requires some reference system and is less transparent when applied purely experimentally to a single isomer.

Although more expensive, NMR spectroscopy is one of the most frequently used techniques in structure elucidation.^15,16^ In particular, ^1^H NMR spectroscopy is a routine technique and must for any chemical laboratory. It has traditionally been applied to closed-shell (diamagnetic) compounds, with notably fewer applications reported for open-shell (paramagnetic) systems.^17^ However, paramagnetic NMR spectroscopy (pNMR) has gained gradually increasing popularity in connection with significant advances in both hardware and methodology and the development of theoretical tools^18,19^ for spectral prediction and interpretation.^20^

pNMR spectroscopy has proven to be an excellent tool for characterizing the structure of paramagnetic systems, including transition-metal complexes.^17,20^ It has been used to study various important coordination compounds containing 3d elements.^21–25^ Another well-established area of application for pNMR is the study of biomolecular systems containing natural or artificially introduced paramagnetic labels.^26^ Recently, applications in supramolecular chemistry and metal cages have been published and reviewed.^27,28^ Besides its use in the study of liquid samples, pNMR is also rapidly developing for use in the solid state in catalysis and energy materials.^29,30^

In addition to the standard NMR parameters for closed-shell systems, such as the NMR shift and the indirect nuclear spin–spin coupling constant,^31,32^ open-shell systems are characterized by an electron–nucleus hyperfine interaction reflected in the hyperfine (Curie) NMR shift.^17,20,33^ Similar to EPR quantities (electronic **g**-tensor and the hyperfine coupling tensor),^34,35^ the hyperfine NMR shift reports subtle details of the coordination geometry of ligands around the central metal atom. This is particularly true for the Fermi-contact contribution to the NMR shift, which originates from the transmission of the spin density from the singly occupied molecular orbital (SOMO)-typically predominantly localized at the transition metal- to the s-orbital of the probed NMR active nucleus.^33^ This “through-bond” mechanism reports the bonding between individual atoms and applies to both molecular^36^ and supramoleculer systems.^37^ In the restricted open-shell SCF scheme, only the atoms involved in the SOMO are exposed to a positive hyperfine shift (*α* spin density) which is denoted as spin delocalization.^38^ The concomitant relaxation (splitting) of doubly occupied MOs in the unrestricted approach results in spin polarization, which is driven by spin exchange interaction. Depending on the SOMO symmetry with respect to the molecular frame, in-plane and out-of-plane transmission pathways can be distinguished^38^ as further discussed in this account for isomers of [Ru(*acac*)Cl_2_L_2_] complexes. Thus, the resulting hyperfine NMR shift can help discriminate the coordination isomerism.^39^

The NMR characteristics of open-shell M^*n*+^(*acac*)_*n*_ compounds have been thoroughly investigated in both the solution^21,40,41^ and the solid state.^22,42,43^ The previously reported common NMR characteristic of the *acac* ligand is the negative value of the ^1^H NMR shift of the methine hydrogen atom. For example, a single *acac* ligand coordinated with Ru(iii) was characterized by ^1^H NMR in K_2_[Ru(*acac*)(*ox*)_2_] with *δ* = −38 ppm.^44^ In our previous theoretical work,^38^ we have also analyzed the hyperfine *trans* influence along the axial path in X-[RuCl_2_NH_3_]-pyridine. There we have identified that direct σ-hyperconjugation delocalization of an unpaired electron can occur only in the high-energy eclipsed conformation, where the Cl–Ru–Cl bond arrangement is parallel to the pyridine ligand. A hyperconjugative interaction occurs when the main spin-carrying orbital on the metal does not form a σ or π bond with a spectator atom, but still overlaps with it in space to enable spin transmission.^45^ In contrast, a perpendicular rotamer with Cl–Ru–Cl orthogonally oriented with respect to the aromatic ring shows a purely π-conjugation delocalization mechanism.

**Fig. 1:**
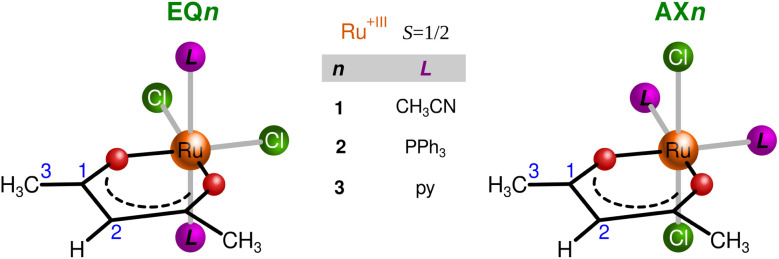
Schematic structures for compounds EQ (equatorial chlorides) and AX (axial chlorides) with pnictogen-based ligands (1: CH_3_CN, 2: PPh_3_, and 3: pyridine).

In this work, we analyze two coordination isomers containing a single bidentate *acac* moiety complemented by two Cl^−^ and two neutral pnictogen-based ligands, as shown in Fig. 1. We have characterized them experimentally in detail and demonstrate here that the two isomers show a fundamentally different distribution of spin density and can be considered models of two distinct hyperfine coupling pathways. These pathways were analyzed using the standard Kohn–Sham DFT approach, which was assumed to be adequate and transparent to describe the propagation of hyperfine effects in the *acac* ligand. Our account demonstrates how the hyperfine interaction can be utilized for the structure elucidation of open-shell metal complexes.

## 2 Results and discussion

### 2.1 Experimental characterization of coordination isomers

**Fig. 2:**
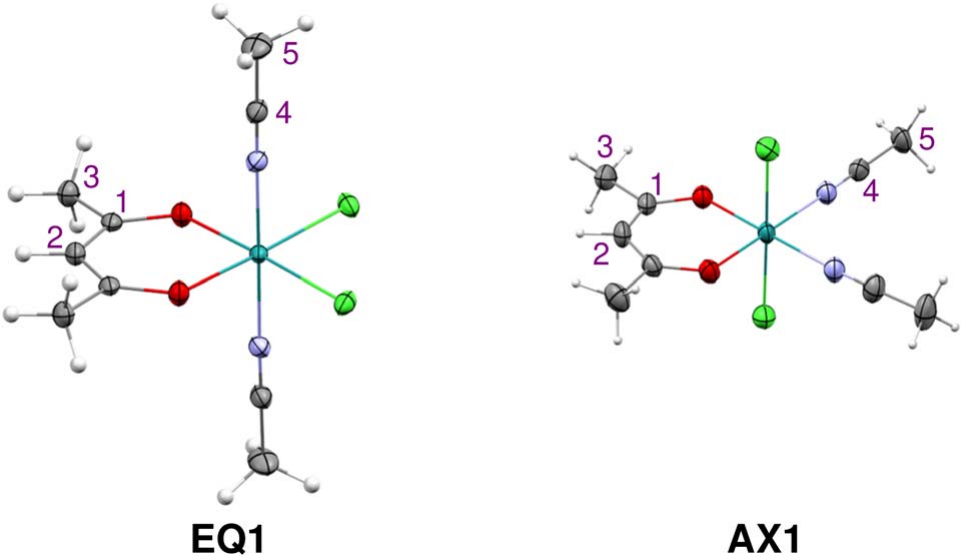
Molecular structures and the atom numbering scheme for compounds EQ1 and AX1 determined by X-ray diffraction analysis. Solvent molecules are omitted for clarity. Detailed parameters of refined structures are summarized in the SI (Table S1).

The two isomers of compound 1 were prepared according to the procedure described in ref. 46 and crystallized as detailed in the SI. The molecular structures of both isomers, EQ1 and AX1, were determined by using X-ray diffraction; see Fig. 2. The compounds adopt a locally octahedral geometry with the *acac* and Cl ligands in the equatorial plane in EQ1 or the *acac* and CH_3_CN ligands in the plane, positioning two Cl ligands in the axial positions in AX1. It is interesting to note that these isomers differ in color, where the solution of EQ1 is red-violet and AX1 is green-blue (for UV-Vis spectra, see Fig. S1 in the SI). This points to different energy and symmetry properties of their frontier molecular orbitals, as will be analyzed and discussed in relation to their UV-Vis spectra and molecular distribution of spin density, which governs hyperfine NMR shifts (*vide infra*).

### 2.2 Paramagnetic NMR spectroscopy

**Fig. 3:**
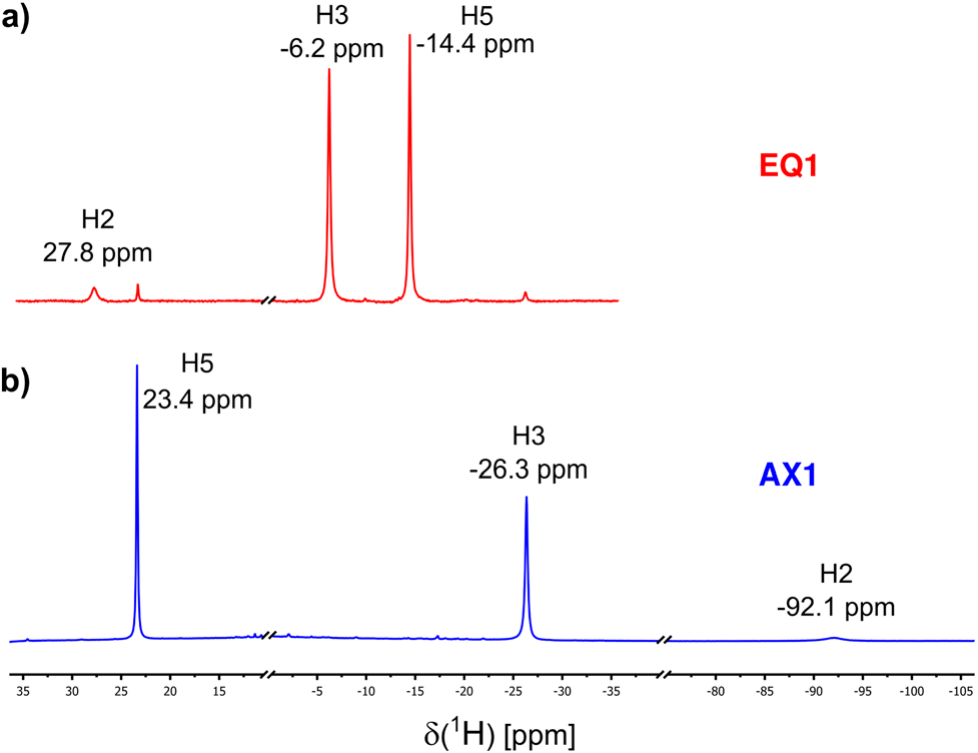
^1^H NMR spectra of (a) EQ1 and (b) AX1 in CDCl_3_, measured at 298 K.

The ^1^H NMR spectra of EQ1 and AX1 are shown in Fig. 3. The methine hydrogen H2 is paramagnetically deshielded in compound EQ1 (*δ*_tot_ = +28 ppm), whereas in AX1 it is highly shielded and found at −92 ppm. This is a great difference that indicates the potential of paramagnetic ^1^H NMR spectroscopy as a sensitive diagnostic tool. The opposite trend in NMR shift has been found for H5, which is shielded in EQ1 but is deshielded in AX1. Both of these observations point to different spin-transmission mechanisms for the two isomers of locally octahedral complexes EQ and AX, as discussed in the following section.

The ^13^C NMR spectra of both coordination isomers that highlight the deshielding effects for C1 in EQ1 and for C4 in AX1 are shown in the SI (Fig. S3). Unfortunately, we were unable to detect methine carbon C2 due to the fast paramagnetic relaxation (compare with Ru(*acac*)_3_ in our previous paper:^41^ the signal of C2 is broader than that of C1).

All experimental ^1^H and ^13^C NMR shifts for EQ1 and AX1 are summarized in Table 1. As indicated in bold in Table 1, the most distinct and diagnostic signals of the *acac* ligand when comparing the two isomers (EQ*vs*. AX) are H2 and C1. Therefore, in further analysis and discussion we focus on the NMR shifts of these two atoms in both isomers. To verify more general validity of the shielding/deshielding trends described above, we also synthesized two coordination isomers containing the PPh_3_ ligand (compound 2 in Fig. 1). The experimental NMR shifts of H2 and C1 atoms in variously substituted isomeric compounds EQ and AX are summarized in Table 2.

**Table 1:** ^1^H and ^13^C NMR shifts measured in CDCl_3_ at 298 K

Atom	EQ1	AX1	Ru(*acac*)_3_^b^
H2	**+27.8**	**−92.1**	−30.4
H3	−6.2	−26.3	−5.6
H5	−14.4	+23.4	—
C1	**+485**	**−261**	+137
C2	a	a	+318
C3	−4	−31	−25
C4	**−120**	**+218**	—
C5	+18	−38	—

a Signals for C2 were not detected.

b Data from ref. 41 (293 K).

**Table 2:** Experimental total and hyperfine ^1^H and ^13^C NMR shifts (in ppm) for compounds EQ1–AX2 measured in CDCl_3_ at 298 K and hyperfine shifts calculated using SO-ZORA/PBE0/TZ2P/COSMO^a^

Isomer	Comp.	H2			C1		
		TOT^exp^	HF^exp^	HF^cal^	TOT^exp^	HF^exp^	HF^cal^
EQ	1	+27.8	+22.3	+45.8	+485	+296	+362
	2	+61.6	+56.1	+77.4	n.d.	n.d.	+459
	3	+27.8	+22.3	+53.2	n.d.	n.d.	+575
AX	1	−92.1	−97.6	−100.9	−261	−450	−529
	2	−80.9	−86.4	−76.2	n.d.	n.d.	−404

a For EPR spectra of compounds EQ2 and AX2, see the SI.

A quantitative comparison of the experimental and calculated NMR shifts can be performed efficiently through hyperfine (Curie) contributions to the NMR shifts determined as a difference between the NMR shift of the paramagnetic Ru compound and its diamagnetic Rh^3+^ (or Ru^2+^) analog, as shown in Table 2. Here, the approximation employing the symmetric diamagnetic Rh(*acac*)_3_ system has been used for two reasons: (i) the experimental inaccessibility of diamagnetic Rh analogs of compounds EQ and AX and (ii) the low stability of Ru coordination isomers, which reduced the effectiveness of measuring the Curie dependence of NMR shifts on temperature and also the accuracy of fitting NMR shifts (see Fig. S6 and Table S5).^47^

Because the trend in experimental hyperfine NMR shifts is very well reproduced by theoretical calculations (see Table 2), we can use the DFT approach to interpret the spin-transmission mechanisms in both isomers. Note that Fermi-contact (FC) contributions dominate the hyperfine NMR shifts for diagnostic atoms H2 and C1 (for contact and pseudocontact NMR contributions, see SI Table S4). Therefore, we interpret the FC hyperfine NMR shifts using standard chemical concepts based on canonical molecular orbitals (MOs).

### 2.3 DFT analysis of frontier molecular orbitals and interpretation of hyperfine couplings

#### 2.3.1 Electronic structure and Kohn–Sham MO analysis

**Fig. 4:**
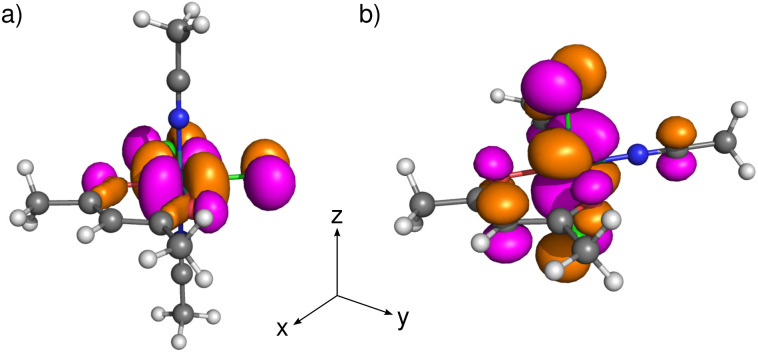
Visualization of HOMOs in the closed-shell Ru^2+^ systems (a) [EQ1]^−^ and (b) [AX1]^−^, at the PBE0/def2-TZVPP/COSMO level, with isosurfaces at 0.03 au. Color coding: H white, C gray, O red, N blue, Cl green.

To analyze the effect of the position of halides on the distribution of spin density in the coordination isomers EQ and AX, we first resorted to closed-shell Ru^2+^ analogs [EQ1]^−^ and [AX1]^−^, formally obtained *in silico* by reducing the central Ru^3+^ atom to Ru^2+^. In these systems, the local octahedral coordination of the central ruthenium atom leads to a d^6^ configuration in the three lower-energy metal d orbitals. While these Ru d orbitals are non-bonding with respect to σ interactions, they are capable of π interactions, leading to low-lying ligand-centered bonding MOs and high-lying metal-centered antibonding MOs.^48^ Strongest π-antibonding interactions are expected for Cl^−^ ligands with the lone pairs of proper symmetry localized on a single atom. In [EQ1]^−^ (the in-plane position of Cl ligands), both chlorides can simultaneously interact with the Ru d_*x*^2^−*y*^2^_ atomic orbital (AO) which consequently dominates the highest lying molecular orbital (HOMO), as shown in Fig. 4a. In [AX1]^−^ (the out-of-plane position of Cl ligands), both chlorides can simultaneously interact with the Ru d_*xz*_ and d_*yz*_ AOs, where, for the former AO, additional antibonding interaction with the *acac* ring must be taken into account. As a result, the HOMO is composed of the metal d_*xz*_ AO in combination with *acac* p_*z*_ and chlorine p_*x*_ AOs, as shown in Fig. 4b. In summary, the HOMO acquires a significant metal-halide π-antibonding character, and its isosurface spatial orientation becomes strongly dependent on the relative arrangements of the chloride ligands within the coordination sphere.

Subsequently, we calculated the electronic structure of open-shell compounds EQ and AX using an unrestricted DFT approach. Subtracting one electron from the closed-shell Ru^2+^ analogs results in splitting of their HOMOs (shown in Fig. 4) into singly occupied molecular orbitals (SOMOs, *α*-spin orbitals) and singly unoccupied molecular orbitals (SUMOs, *β*-spin orbitals) in Ru^3+^ systems. The stabilization of SOMO levels below several formally doubly occupied MOs in these compounds (see Fig. S11) arises from stronger intra-orbital stabilization of *α* spin-orbitals than the energy lowering of the spatially disjoint HOMO−1 and HOMO−2 orbitals.^49^ However, this energy stabilization has only a small influence on the qualitative composition of MOs and, therefore, SOMOs (and SUMOs) of EQ and AX closely resemble HOMOs of the corresponding closed-shell analogs discussed above. The consequences of the change in MO composition for the spectroscopic parameters are discussed in the following subsections.

#### 2.3.2 Visible bands in UV-Vis spectra

To analyze the diagnostic potential of UV-Vis spectroscopy in characterizing the coordination isomerism indicated above, we used a TD-DFT approach^50^ to predict UV-Vis spectra for the isomers EQ and AX with a series of ligands shown in Fig. 1 and an analog of compound 1 obtained by substituting bromides for chlorides (1-Br).

**Fig. 5:**
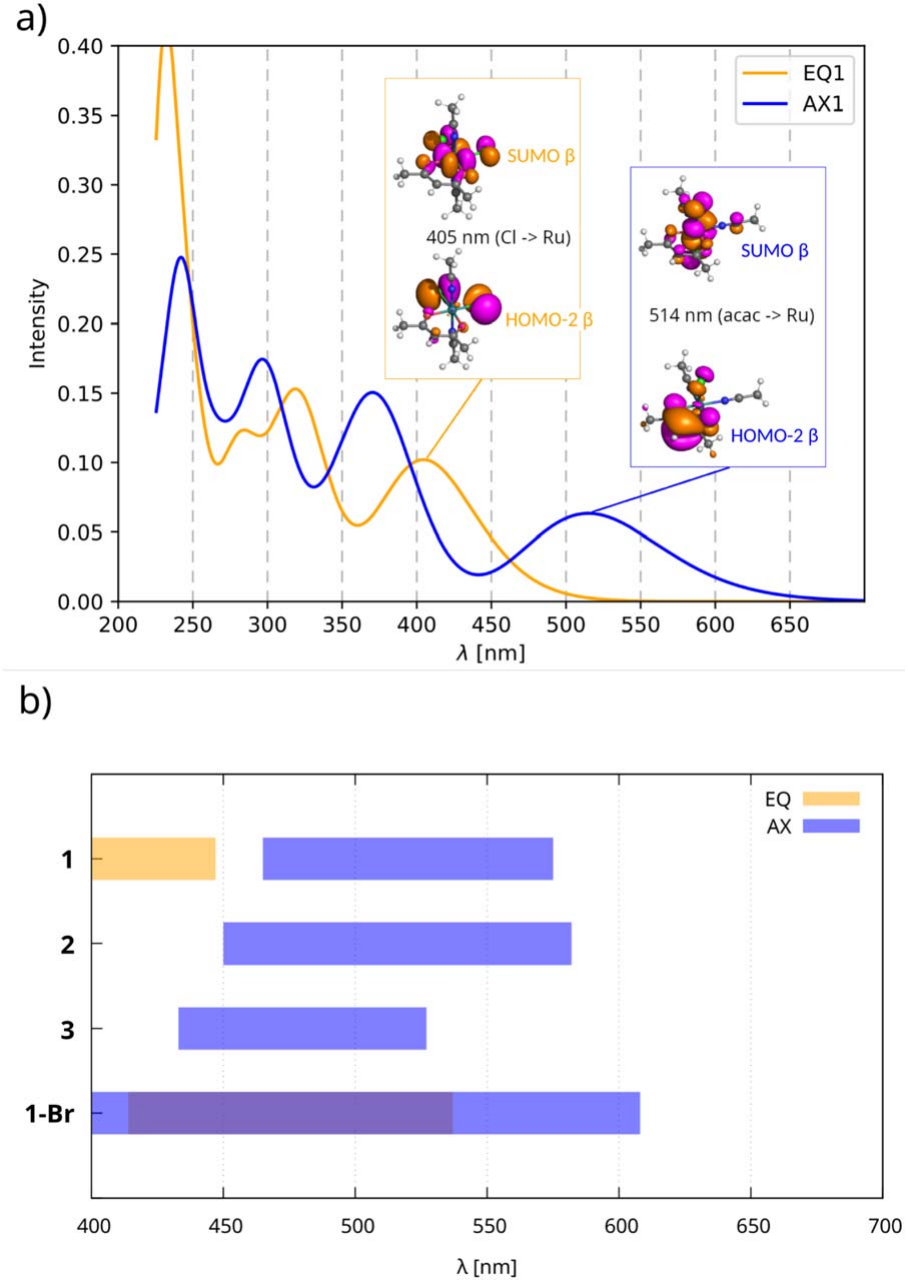
(a) UV-Vis spectra of EQ1 and AX1 simulated by the TD-DFT approach at the UKS/PBE0/def2-TZV2P/COSMO(chloroform) level with 0.5 eV Gaussian broadening. Note the ligand-to-metal charge-transfer (LMCT) from ligand-based orbitals to the transition metal (SUMO). (b) Ranges of visible absorption bands calculated for both isomers bearing various halogen and pnictogen ligands. Note that the lowest-energy absorption of EQ2 and EQ3 is predicted to be below 400 nm.

The predicted bands in the visible region are governed by excitations from the occupied space to SUMOs (Fig. 5a). The occupied MOs involved in these excitations are dominated by non-bonding in-plane AOs (p_*x*_/p_*y*_) of chloride ligands in compounds EQ and out-of-plane AOs (p_*z*_) of the *acac* ligand in compounds AX, while SUMOs are more metal-centered. Thus, the ligand-to-metal charge-transfer (LMCT) mechanism gives rise to the long-wavelength bands observed in the UV-Vis spectra of both isomers. We recall that the shapes of the SUMOs closely resemble those of the HOMOs of their closed-shell analogs shown in Fig. 4.

Although this technique seems reliable for characterizing isomers if both compounds have been synthesized and measured, visible bands are greatly influenced by the nature of coordinating ligands, as shown in Fig. 5b. Therefore, the experimental UV-Vis approach (see Fig. S1 and S2 in the SI) is rather equivocal if only a single isomer is formed.

#### 2.3.3 Interpretation of hyperfine NMR shifts

As indicated in Section 2.2, ^1^H and ^13^C NMR shifts of some atoms of the *acac* ligand are very sensitive indicators of coordination isomerism. To reveal the nature of the differences in NMR shieldings between the two isomers, we performed DFT calculations and analyzed hyperfine NMR shifts and isotropic hyperfine coupling constants (*A*_iso_) in terms of canonical MOs.

**Fig. 6:**
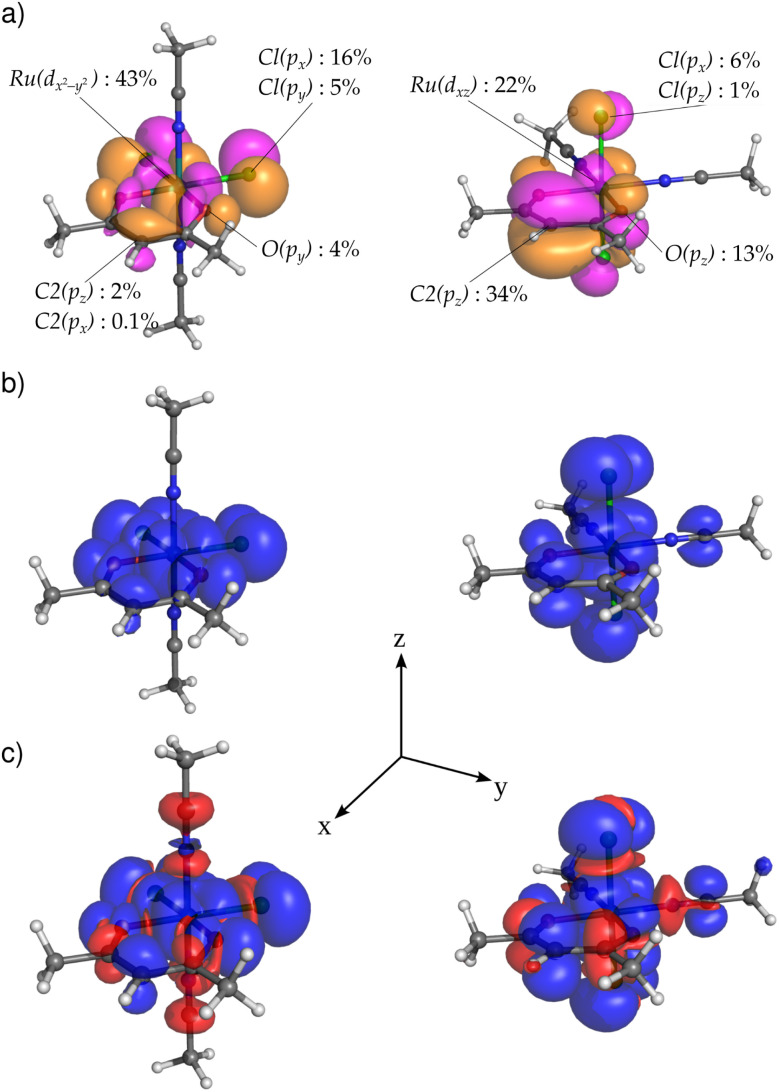
Visualization of (a) SOMO isosurfaces with labels for selected important contributions from the AOs (>1%, except for H2), and (b and c) spin density for compounds EQ1 (left) and AX1 (right) calculated using (b) restricted and (c) unrestricted approaches at the PBE0/def2-TZVPP/COSMO level, with isosurfaces at 2.5 × 10^−4^ a.u.; spin density *α* in blue and *β* in red.

For any well-defined electronic doublet system, hyperfine NMR shifts can be calculated from the molecular electronic **g**-tensor and atom-specific **A**-tensors.^18,51,52^ Furthermore, the dominant Fermi-contact contributions to the hyperfine NMR shifts can be transparently linked to the underlying Fermi-contact mechanisms of hyperfine couplings.^33^ In the non-relativistic regime and negligible spin-dipolar contribution, the isotropic hyperfine coupling constant *A*_iso_ is directly related to the imbalance between *α* and *β* electron density residing at the nucleus. Therefore, we focus our following analysis and discussion on the link between SOMO compositions and molecular distribution of spin density shown in Fig. 6, atomic spin populations, and hyperfine coupling constants. The principal difference in the transmission of the spin density from the metal to the *acac* ligand in EQ*versus*AX arises from the different orientations of the SOMO isosurfaces shown in Fig. 6a.

In compound EQ1, the spin density propagates mainly through a delocalization mechanism in the *xy* plane. As a result, significant *α* spin density (shown in blue in Fig. 6b) is obtained not only for both in-plane Cl ligands, but also for all carbon atoms of the *acac* ring, finally reaching hydrogen H2. This gives rise to a positive hyperfine ^1^H NMR shift for H2 (see Table 1). Although spin polarization generates some *β* spin population in the C1 p_*z*_ AO (*cf*. *β* spin density shown in red in Fig. 6c), it cannot overbalance the effect of direct in-plane delocalization, resulting in a total positive hyperfine NMR shift also for C1 (see Table 1).

In compound AX1, with Cl ligands above and below the *acac* ring, the *acac* ligand AOs contribute to the SOMO mostly in the out-of-plane manner (*e.g*., 34% of the C2 p_*z*_ AO in the SOMO). For this arrangement, direct spin delocalization in the out-of-plane π-space is an important factor due to π-electron conjugation, which generates a positive spin population of the C2 2p_*z*_ AO. As a result of the spin-polarization mechanism described by McConnell's relation,^53^ a negative spin density (*β*, a small red ball in the right part of Fig. 6c) and a negative hyperfine NMR shift (Table 1) are obtained for H2. The spin-polarization mechanism thus gives rise to negative hyperfine shifts for C1 and H2 atoms.

In brief, the local predominance of *α*-spin density at H2 of EQ1 results in a hyperfine NMR deshielding effect, whereas the local predominance of *β*-spin density at H2 of AX1 results in a hyperfine NMR shielding effect.^38^ This leads to a notable, easily measurable difference between the two complexes, as summarized in Table 2.

For the strictly planar geometry of the EQ1 isomer (transition state), symmetry forbids any contribution of the C2 p_*z*_ AO to the SOMO, resulting in zero spin population in this orbital (see Table S6 in the SI). In contrast, the slightly distorted geometry of the *acac* ligand observed in the experimental X-ray structure (and reproduced in DFT-optimized geometries) relaxes this symmetry restriction and permits a small p_*z*_(C2) contribution to the SOMO. The resulting delocalized spin population in the p_*z*_ orbital generates, according to McConnell's relation, a small spin density of opposite sign at H2, leading to a slight reduction in the experimental hyperfine shift of H2 relative to that for the idealized planar transition-state geometry due to the competing σ and π spin-transmission pathways.

For isomers of compound 1, we can also observe an isomer-dependent spin distribution in the *acn* ligand. In AX1, atom C4 shows a positive p_*z*_ spin population due to the out-of-plane π-conjugation delocalization from the metal center and a polarization-induced negative spin density in the equatorial plane (Fig. 6c). In contrast, atom C4 in EQ1 bears a completely negative spin population resulting exclusively from the spin-polarization mechanism (delocalization from the in-plane SOMO is symmetrically forbidden). The opposite spin polarity of the triple bond is reflected in the inverted hyperfine shifts of the methyl groups (higher absolute value observed for AX1, see Table 1).

In summary, the variation in SOMO composition between the two coordination isomers dictates the spin-transmission mechanism, classified as in-plane σ-hyperconjugation or out-of-plane π-conjugation delocalization,^38^ leading to pronounced hyperfine shifts that can be directly probed by ^1^H NMR spectroscopy.

## 3 Conclusions

The hyperfine NMR shift is shown to be an extremely sensitive reporter of the change in the electronic structure between equatorial and axial coordination isomers of a Ru(iii) mono-*acac* compound. In EQ and AX isomers, we have identified fundamentally different transmission mechanisms of spin density: in-plane σ-hyperconjugation delocalization *versus* out-of-plane π-conjugation delocalization (accompanied by spin polarization), respectively. The former pattern appears to be unique in the context of pNMR data for Ru(iii)–*acac* complexes.

As demonstrated herein, paramagnetic NMR emerges as a simple, unambiguous analytical tool for characterizing coordination isomerism. Notably, a transparent link is revealed between changes in coordination geometry and the electronic structure and pronounced variations in spin density and NMR response. We also identified a substantial effect of non-planarity of the *acac* ring on the magnitude of the hyperfine shift of the H2 atom. Therefore, a systematic and comprehensive investigation of the relationships among geometry, the nature of molecular orbitals, and Curie NMR shifts is currently underway in our laboratory.

## Author contributions

DC: theoretical investigation, data curation, visualization, writing – original draft; JC: experimental investigation, data curation, visualization; MM: theoretical investigation, supervision; YY: theoretical investigation; JN: conceptualization, methodology, theoretical investigation, data curation, supervision, resources, writing – original draft, review and editing; RM: conceptualization, project administration, supervision, resources, funding, writing – original draft, review and editing.

## Conflicts of interest

There are no conflicts to declare.

## Supplementary Material

SC-017-D5SC09905F-s001

SC-017-D5SC09905F-s002

## Data Availability

The single-crystal X-ray diffraction data for compounds EQ1 (CCDC No. 2514733) and AX1 (CCDC No. 2514734) are available at the Cambridge Crystallographic Data Center. The NMR spectra have been deposited at Mendeley Data and can be accessed at https://doi.org/10.17632/dz4gdnx7h9.1. The computational results are available in the ioChem-BD repository^54^ and can be accessed at https://doi.org/10.19061/iochem-bd-6-622. CCDC 2514733 (EQ1) and 2514734 (AX1) contain the supplementary crystallographic data for this paper.^55*a*,*b*^ Supplementary information (SI) is available. See DOI: https://doi.org/10.1039/d5sc09905f.
