# Supplementary material for: Unraveling the coordination isomerism by ligand hyperfine NMR shifts
Source: Chem Sci. 2026 Mar 4;17(18):9042–8. doi: 10.1039/d5sc09905f (PMC12990213; doi:10.1039/d5sc09905f)
Supplement: SC-017-D5SC09905F-s001 [file SC-017-D5SC09905F-s001.pdf]

Supplementary Information (SI) for Chemical Science.
This journal is © The Royal Society of Chemistry 2026

ELECTRONIC SUPPLEMENTARY INFORMATION

Unraveling the coordination isomerism by ligand hyperfine NMR shifts

Dora Cidlinská[‖,†,‡] Jan Chyba[‖,†,‡] Markéta Munzarová,[‡] Yevgen Yurenko,[†,¶] Jan Novotný,[*,†,‡,¶] and Radek Marek[*,†,‡,¶]

†*CEITEC - Central European Institute of Technology, Masaryk University, Brno, Czechia*

‡*Department of Chemistry, Faculty of Science, Masaryk University, Brno, Czechia*

¶*National Center for Biomolecular Research, Faculty of Science, Masaryk University, Brno, Czechia*

E-mail: jan.novotny@ceitec.muni.cz; radek.marek@ceitec.muni.cz

[‖] These two authors contributed equally.

1 Methods

1.1 Preparation and structure characterization

Ruthenium(III) chloride hydrate (99.9% Ru) was obtained from *abcr* GmbH, acetylacetone (99%) was purchased from *TCI Europe* N.V, triphenylphospine (99%) was purchased at *Fluorochem* Ltd. All nondeuterated solvents - acetonitrile, dichlormethane, diethylether, ethanol, and pyridine, were of *p.a.* grade and were used as received (Lachner). Deuterated chloroform (99.8% D) stabilized with silver foils was obtained from Eurisotop and used to prepare NMR samples.

The *equatorial* (**EQ**) and *axial* (**AX**) isomers of the coordination compound $[Ru(acac)(L)_2Cl_2]$ (L = CH_3CN, PPh_3, pyridine, see **Fig. 1**) were prepared according to previously published synthetic procedures.[1,2] The product **EQ** was formed and filtered out after cooling the concentrated reaction mixture at -20°C, the second compound **AX** was precipitated from the residual mother liquor by adding diethylether. Both products were purified by recrystallization from a dichloromethane/ diethylether mixture. In the case of the $[Ru(acac)(py)_2Cl_2]$ complex, only the equatorial form (**EQ**) was successfully isolated from reaction mixture.

The monocrystals of coordination isomers **EQ1** and **AX1** were grown at room temperature by slow evaporation of solvent from concentrated solution of the complex in dichlormethane.

1.2 Single-crystal X-ray diffraction

The diffraction data were collected on a Rigaku Mi croMax-007 HF rotating anode CCD diffractometer. The structures were solved by direct methods and refined by full matrix least-squares methods using the SHELXT software package.[3]

Crystal data and structure refinement parameters are summarized in **Table S1**.

Table S1: Selected crystallographic data for the Ru(III) coordination compounds **EQ1** and **AX1**

	**EQ1**	**AX1**
CCDC No	2514733	2514734
chemical formula	$C_{11}H_{17}Cl_6N_2O_2Ru$	$C_{10}H_{15}Cl_4N_2O_2Ru$
formula weight	523.03	438.11
crystal system	Monoclinic	Triclinic
space group	*I 2/a*	*P*-1
a (Å)	15.33347(12)	7.70943(12)
b (Å)	8.93579(7)	8.63316(11)
c (Å)	15.08676(13)	25.6307(2)
α (deg)	90	86.5353(9)
β (deg)	108.3791(9)	86.7221(10)
γ (deg)	90	81.2162(12)
V (Å^3)	1961.69(3)	1680.82(4)
Z	4	4
D_{calcd} (g cm^{-3})	1.771	1.731
μ (mm^{-1})	14.048	13.404
measured/unique reflections	12194/ 2075	39228/6981
data/parameters/restraints	2075/104/0	6981/351/0
R_1/wR_2 [I>2$\sigma(I)$]	0.0218/0.0592	0.0352/0.0965
R_1/wR_2 [all data]	0.0219/0.0592	0.0369/0.0977
GoF	1.125	1.076
$\Delta\rho^{max}/\Delta\rho^{min}$ (e Å^{-3})	0.772 / -0.476	0.648 / -1.461

1.3 UV-VIS analysis

UV-Vis spectra were acquired using a Biochrom WPA Lightwave II spectrophotometer equipped with xenon light source and the CCD array detector. The solutions of the samples were filled to quartz cuvettes and measured in a wave scan mode ranging from 250 to 850 nm.

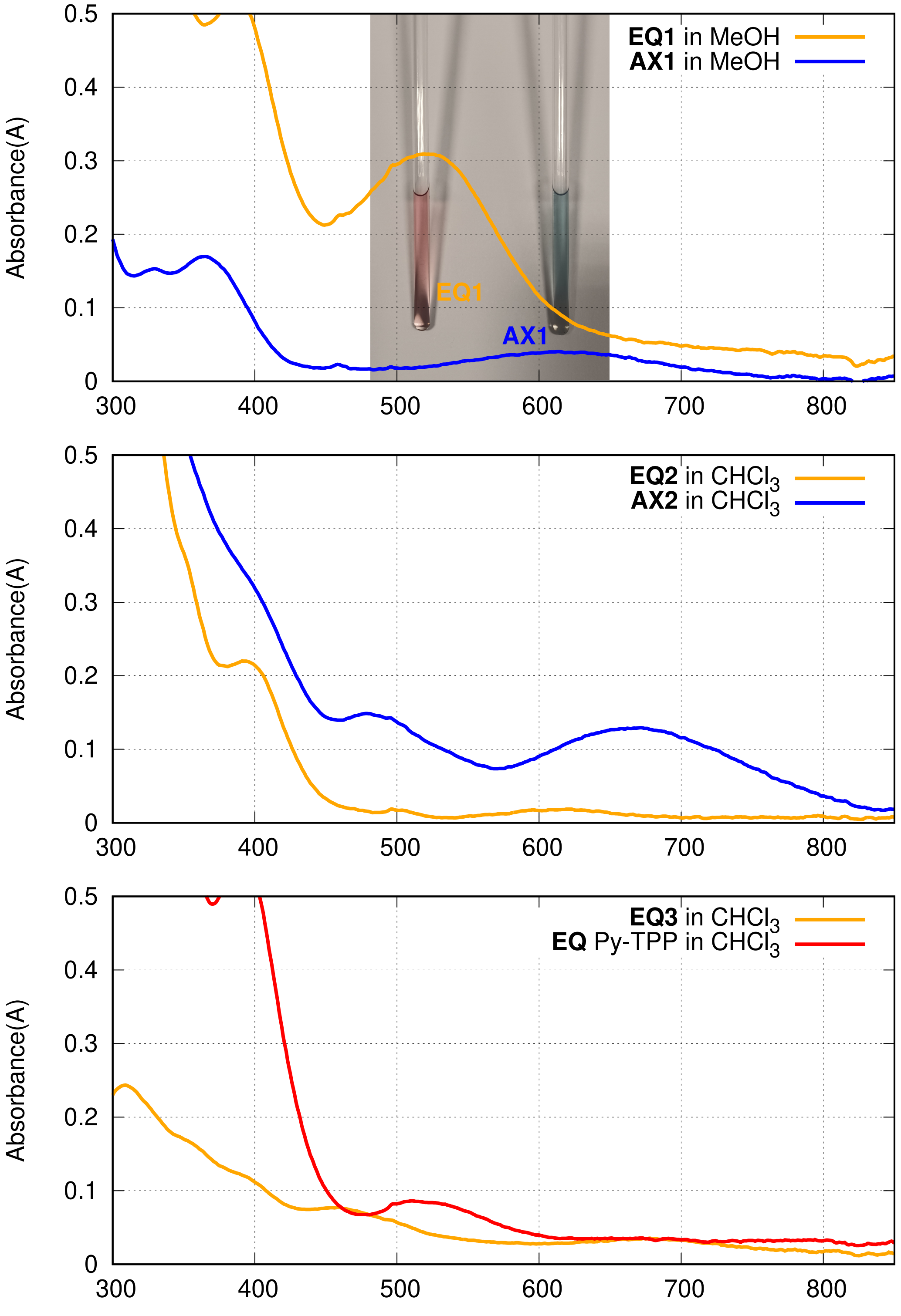

Fig. S1: Experimental UV-VIS absorption spectra of experimentally available complexes: (*top*) **EQ1** (red-violet solution) and **AX1** (green-blue solution) measured in methanol, (*middle*) **EQ2** and **AX2** in chloroform, and (*bottom*) **EQ3**.

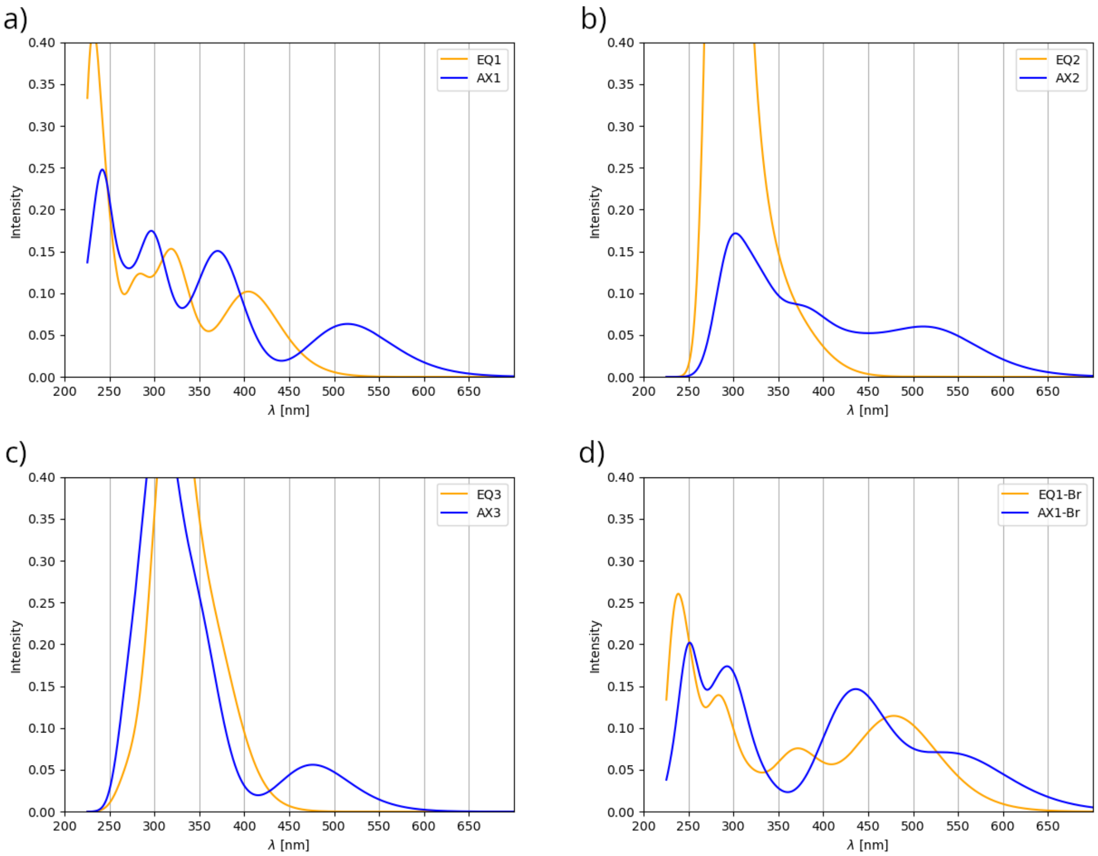

Fig. S2: Simulated UV-VIS absorption spectra of complexes: (*a*) **EQ1** and **AX1**, (*b*) **EQ2** and **AX2**, (*c*) **EQ3** and **AX3**, and (*d*) **EQ1**-Br and **AX1**-Br, all calculated using TD-DFT/PBE0/def2-TZVPP/CPCM(chloroform) approach. Absorption bands were plotted as Gaussian functions with broadening factor 0.5 eV.

1.4 NMR spectroscopy

^{1}H and ^{13}C NMR spectra were recorded on Bruker Avance III HD 700 MHz and Bruker Avance III HD 600 MHz spectrometers, at 298.2 K, unless stated otherwise. Concentrated samples were prepared by dissolving 20-30 mg of metallo-complex in 0.5 mL of $CDCl_3$. ^{1}H and ^{13}C NMR spectra were referenced internally to residual solvent peaks and the NMR shifts are reported relative to tetramethylsilane, TMS ($\delta = 0$ ppm).

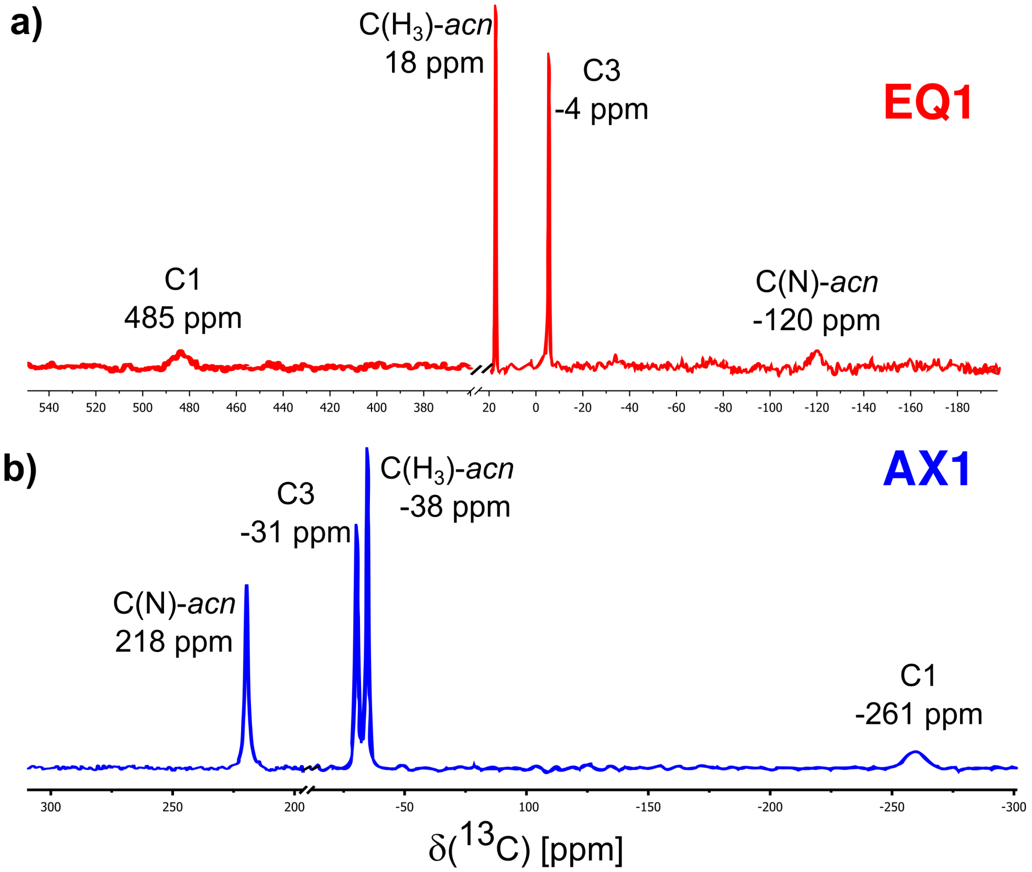

Fig. S3: ^{13}C NMR spectra of a) **EQ1** and b) **AX1** measured in $CDCl_3$.

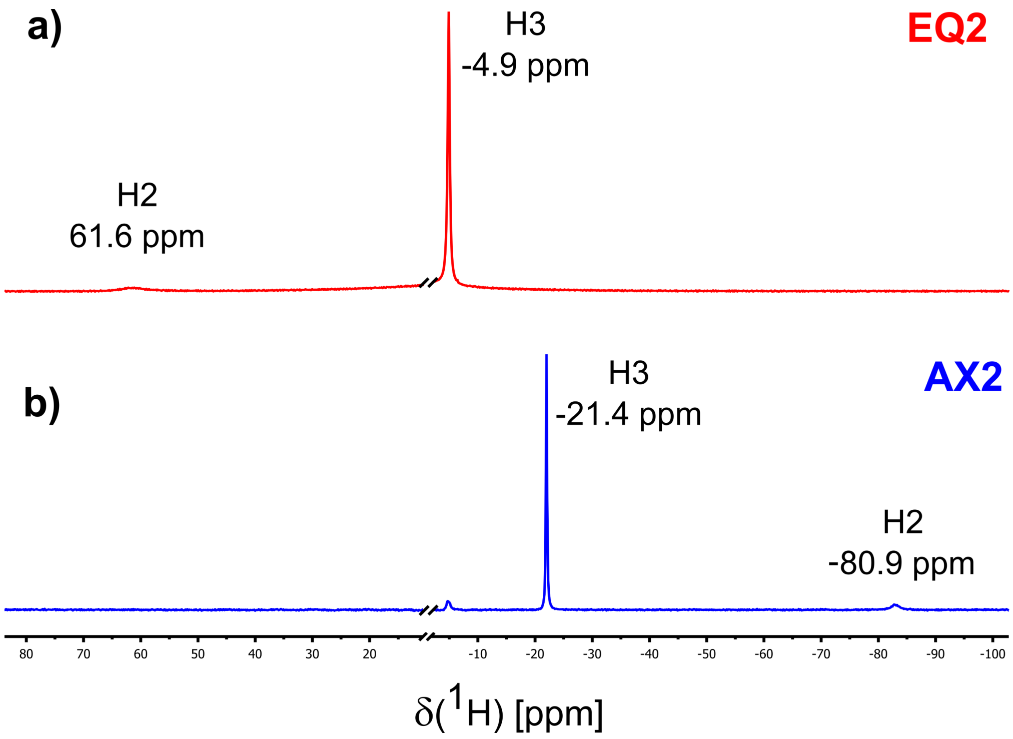

Fig. S4: ^{1}H NMR spectra (acac ligand region) of a) **EQ2** and b) **AQ2** measured in $CDCl_3$.

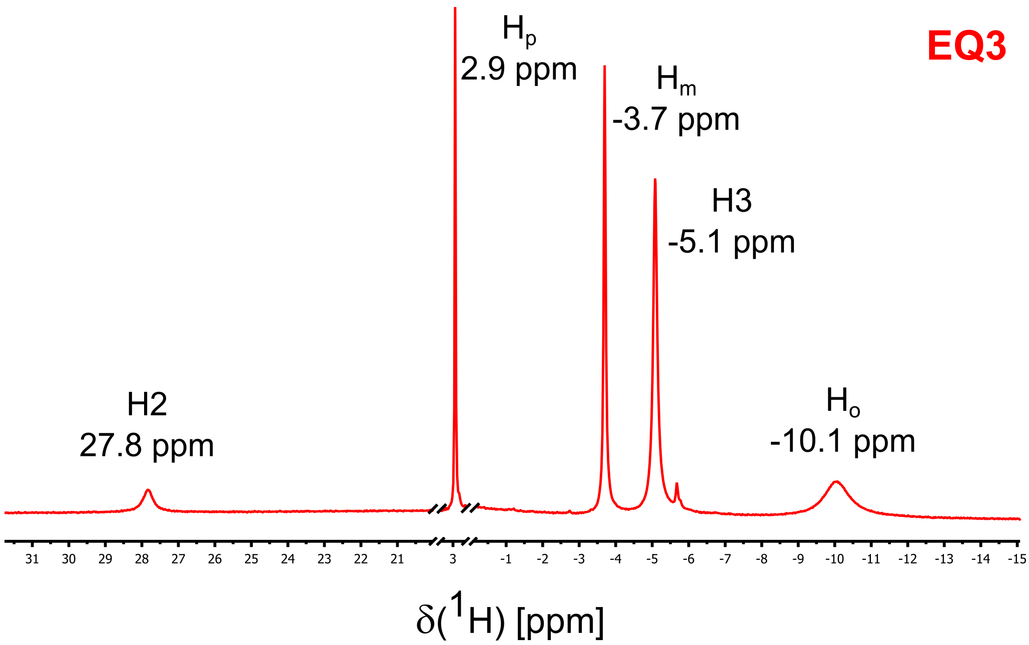

Fig. S5: ^1H NMR spectrum of **EQ3** measured in $CDCl_3$.

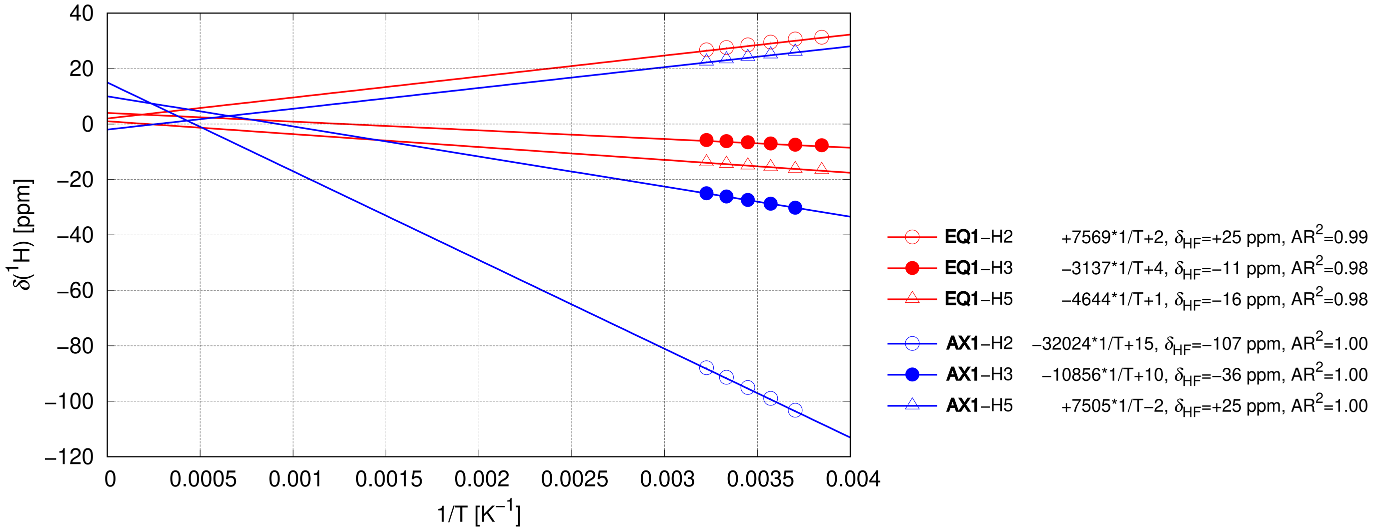

Fig. S6: Curie plots (1/T) of ^1H NMR shifts measured for **EQ1** and **AX1** in $CDCl_3$ in the temperature range 260-310 K.

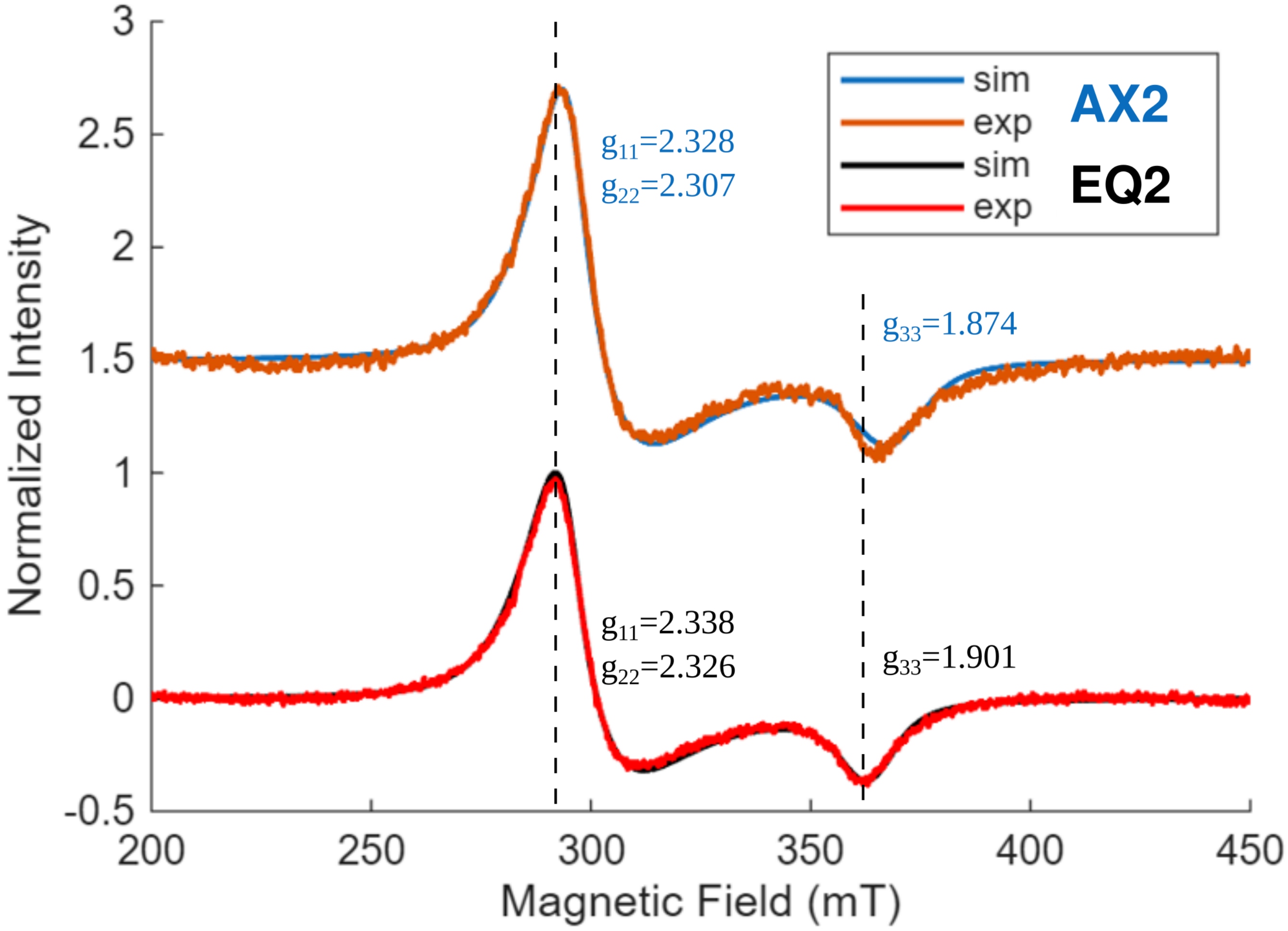

Fig. S7: EPR spectra of powder samples of **EQ2** (*bottom*) and **AX2** (*top*) recorded using benchtop X-band 9.75GHz Bruker EMXnano spectrometer at laboratory temperature with fitted values of principal components of g-matrix. (Default setting parameters for transition metals mode in *Xenon* operating software were used - magnetic field range of 190—450 mT, 0.3162 mW power, and 60s scans.) The spectra were processed in MATLAB using the EasySpin module with following set of parameters: homogeneous linewidth = 11.8/11.9 mT, inhomogeneous linewidth = [0 793 203]/[0 807 303] % in MHz. Corresponding isotropic values for **EQ2** and **AX2** are 2.189 and 2.170, respectively.

1.5 Quantum chemical calculations

The geometries of both isomers of [Ru(*acac*)(N/P)$_2$Cl$_2$] were optimized at the UKS/PBE0/def2-TZVPP/ECP level in Orca 6.0[4] followed by frequency calculation to confirm a local minimum and finally by the analysis of spin density and hyperfine coupling constants (A-tensor). TD-DFT methodology was employed to obtain UV-VIS transitions. pNMR shifts computed at the UKS/SO-ZORA/PBE0/TZ2P/COSMO(chloroform) level were extracted from ADF 2024[5] based on geometries reoptimized using one-component ZORA setup *ibidem*. Hyperfine shifts were correlated with Mulliken atomic spin populations from ZORA densities. Additionally, the DLPNO-HFC2/CCSD (cc-pwCVTZ/Ru:def2-TZVPP, COSMO) approach[6] was tested to estimate the impact of electron correlations on the A-tensor values.

2 Computational analysis

Table S2: Interatomic distances (in Å), angles and dihedral angles (both in °) in various geometries of [Ru(*acac*)(*acn*)$_2$Cl$_2$] isomers, namely: X-ray, Orca, ADF.

Isomer	MODEL	Ru−O	Ru−N/P	Ru−Cl	Ru−O−C1	Ru−O−C1−C2
	X-ray	2.016	2.020	2.331	127	6
**EQ1**	ORCA	2.018	1.996	2.328	127	10
	ADF	2.021	2.004	2.332	126	10
	X-ray	1.997	2.049	2.327	124	3
**AX1**	ORCA	1.992	2.009	2.337	124	0
	ADF	1.997	2.018	2.341	124	0
**EQ2**	ADF	2.024	2.423	2.345	128	0
**AX2**	ADF	2.054	2.406	2.339	126	3
**EQ3**	ADF	2.014	2.084	2.337	127	5

As seen in **Table S2**, when the positions of the Cl^- and CH_3CN ligands are switched, the geometry of the *acac* ring changes slightly.

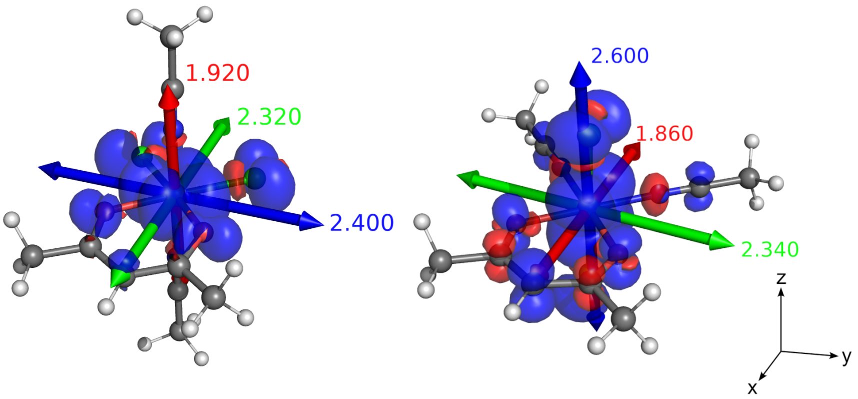

Fig. S8: Molecular g-tensors of the **EQ1** and **AX1** complexes, computational setup SO-ZORA/PBE0/TZ2P. Corresponding g-isovalues are: 2.21 and 2.27. Note the largest positive component Δg_{zz} in **AX1** molecule, originating from the efficient magnetic coupling of β-SUMO(d_{xz}) with doubly-occupied β-MO(d_{yz}).

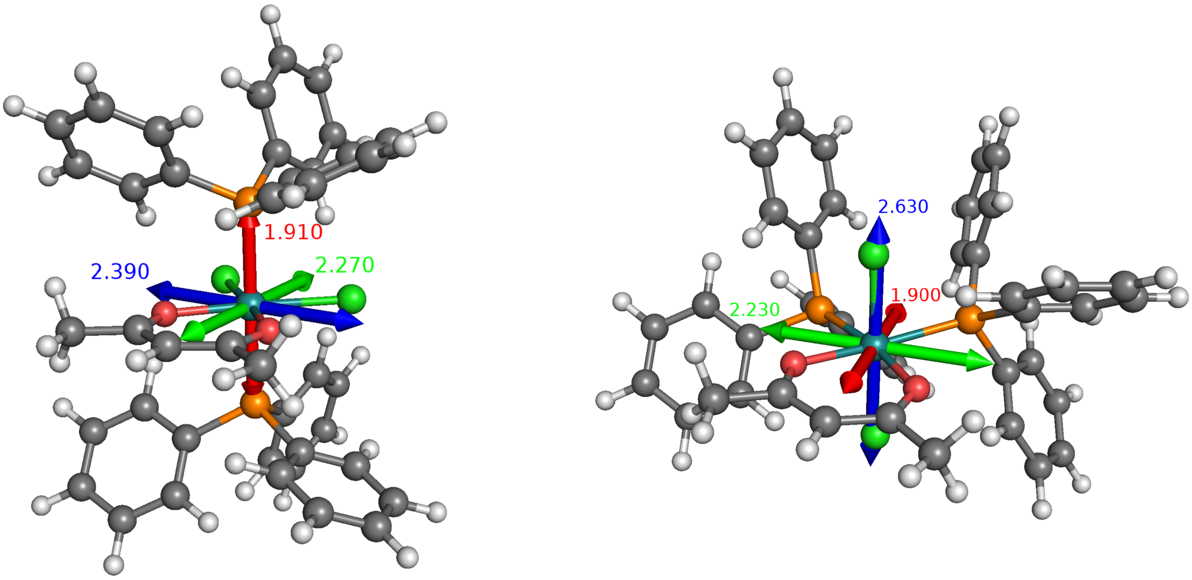

Fig. S9: Molecular g-tensors of the **EQ2** and **AX2** complexes, computational setup SO-ZORA/PBE0/TZ2P/COSMO. Corresponding g-isovalues are: 2.19 and 2.26.

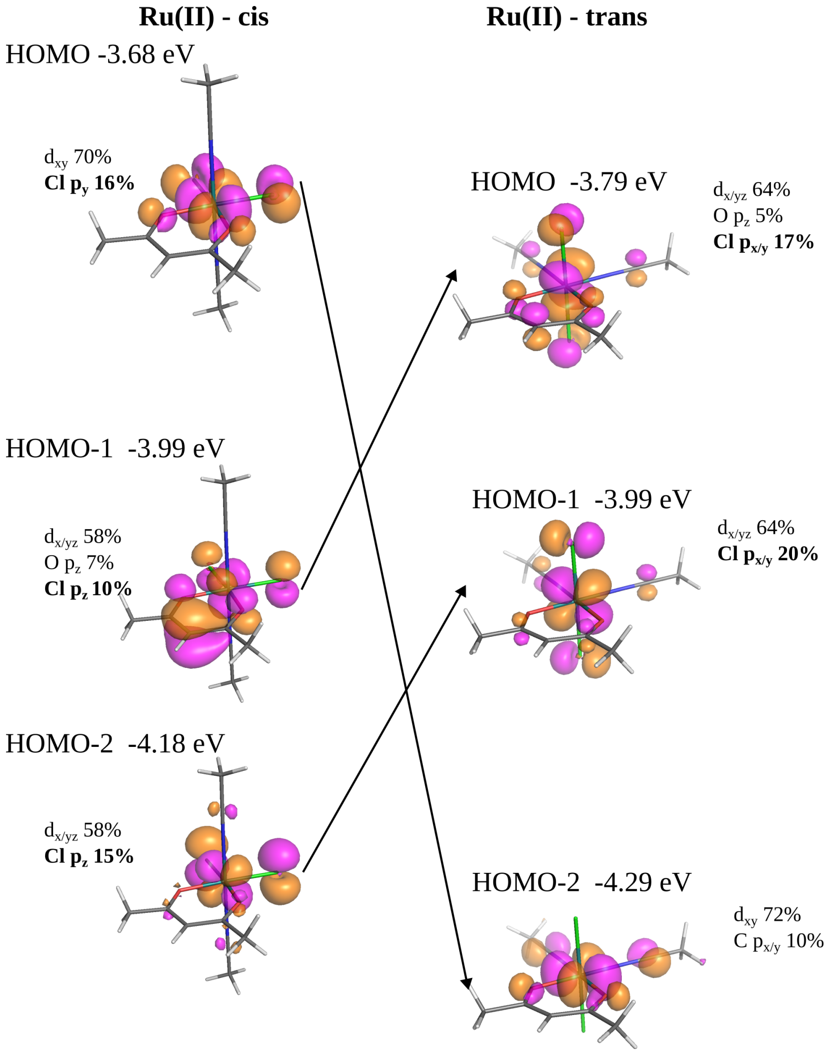

Fig. S10: Visualization of three highest occupied canonical MOs for diamagnetic complexes [**EQ1**]$^-$ and [**AX1**]$^-$ at the PBE0/def2-TZVPP/COSMO level. Selected contributions of AOs obtained from Löwdin population analysis are shown. The energy switch of in-plane and perpendicular d-based oribital between [**EQ1**]$^-$ and [**AX1**]$^-$ can be rationalized by means of degree of antibonding interaction of Ru(II) with Cl lone pairs.

In *equatorial* isomer, the HOMO is formed by a strongly destabilizing out-of-phase combination of equatorial d_{xy} and $p_{x/y}$. This interaction is not possible in *axial* arrangement (that is why the in-plane MO descends to HOMO-2). In contrast, the perpendicular interaction of $d_{x/yz}$ with p_z of Cl in *cis* configuration shows less antibonding character due to the diagonal direction of both d-orbitals relative to Ru-Cl bonds, while in the case of *axial* isomer both perpendicular d orbitals form a strong antibonding interaction with axial Cl ligands.

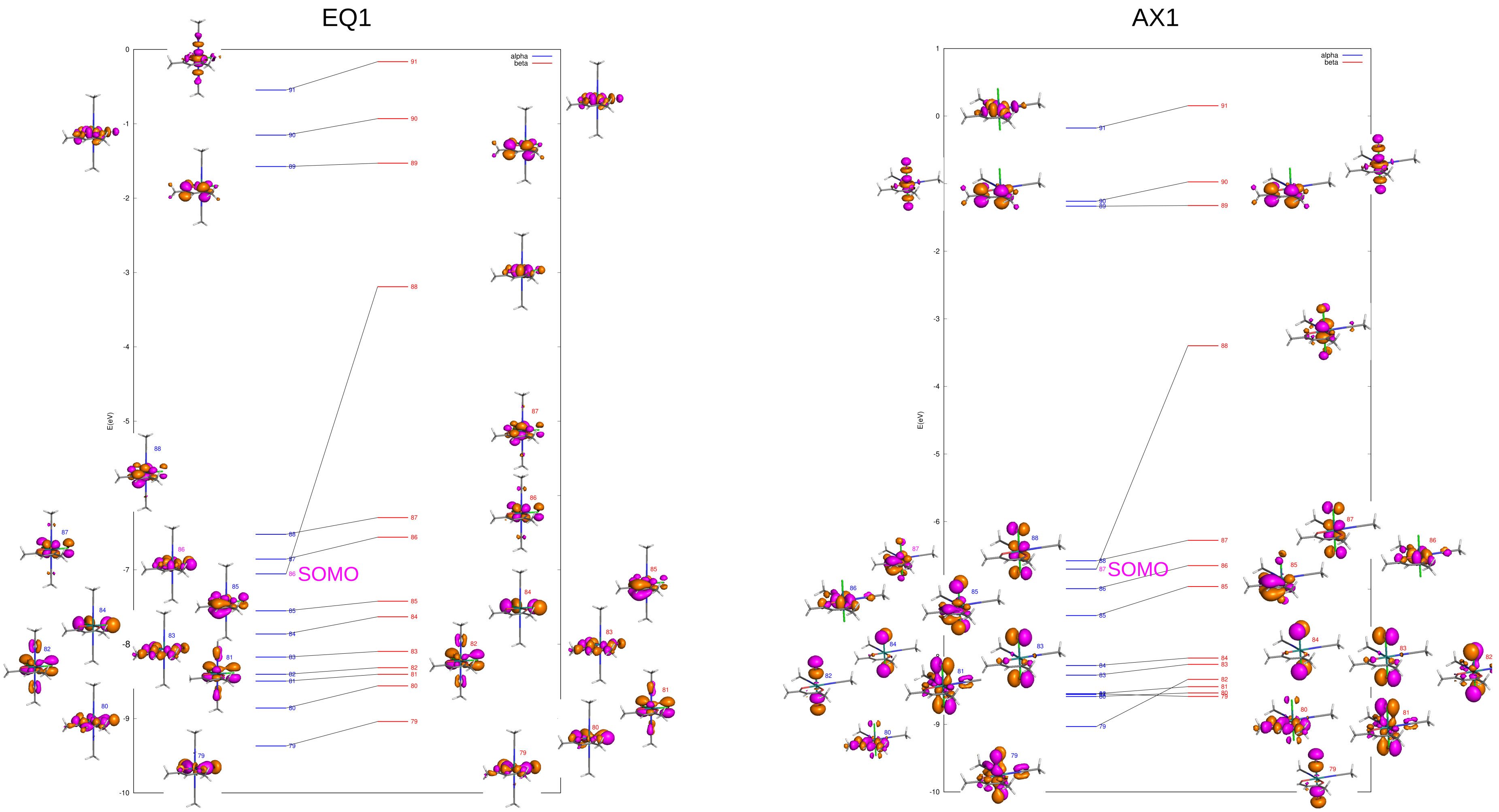

Fig. S11: Energy diagram of frontier MOs of **EQ1** (*left*) and **AX1** (*right*) calculated at the ZORA/PBE0/TZ2P/COSMO level of theory.

2.1 Propagation of hyperfine NMR effects

Table S3: Hyperfine NMR shifts (in ppm) for **EQ1** and **AX1** calculated using the SO-ZORA approach (PBE0/TZVPP, chloroform, fully optimized geometries if not stated otherwise) at 298 K.

Isomer	Atom	PBE0 -25% *	PBE0 -25%	PBE0 -25% †	PBE0-25% QZ4P-J	PBE0 -40%	CAM-B3LYP	EXP ‡
**EQ1**	H2	+77	+47	+46	+46	+35	+50	+22.7
	H3(Me-*acac*)	−8	−13	−13	−12	−12	−13	−8.3
	H5(Me-*acn*)	−24	−26	−25	−24	−24	−26	−16.4
	C1	+668	+369	+368	+389	+383	+359	+296
	C2	+367	+510	+499	+510	+419	+553	n.d.
	C3(Me-*acac*)	−82	−45	−46	−43	−59	+4	−31
	C4(N)-*acn*	−326	−325	−317	−310	−331	−298	−238
	C5(Me-*acn*)	+29	+33	+30	+33	+23	+28	+16
**AX1**	H2	−91	−103	−102	−87	−89	−94	−97.9
	H3(Me-*acac*)	−44	−46	−46	−42	−34	−45	−28.6
	H5(Me-*acn*)	+38	+42	+40	+37	+19	+38	+21.4
	C1	−503	−540	−530	−477	−426	−505	−450
	C2	+418	+472	+458	+376	+269	+450	n.d.
	C3(Me-*acac*)	−50	−46	−50	−51	−38	−48	−58
	C4(N)-*acn*	+62	+166	+157	+63	+133	+160	+100
	C5(Me-*acn*)	−55	−56	−53	−55	−46	−48	−40

Table S4: Comparison of A_{iso} (in MHz) and hyperfine NMR shifts (in ppm), with their contact and pseudocontact contributions, of *acac* atoms at 298 K. Computational setup: SO-ZORA/PBE0/TZ2P/COSMO(chloroform).

	Atom	A_{iso}	$\delta^{HF}(tot)$	$\delta(FC)$	$\delta(PC)$		A_{iso}	$\delta^{HF}(tot)$	$\delta(FC)$	$\delta(PC)$
**EQ1**	H2	+1.52	**+46**	+45	+1	**AX1**	−3.09	**−102**	−94	−8
	H3	−0.50	**−13**	−15	+2		−1.46	**−46**	−45	−1
	C1	+2.97	**+368**	+351	+17		−4.07	**−530**	−493	−37
	C2	+4.34	**+499**	+512	−13		+3.43	**+458**	+415	+43
	C3	−0.40	**−46**	−47	+1		−0.39	**−50**	−48	−2

*Geometry from an X-ray analysis, hydrogen atoms optimized, PBE0/TZVPP, chloroform.

†Geometry from ADF ZORA/PBE0/TZ2P, chloroform.

‡Experimental values obtained with respect to diamagnatic reference $Rh(acac)_3$ (H2 5.5 ppm, H3, C1 189.[7] Signals of coordinated *acn* referenced to free solvent molecule: H5 1.97 ppm, C4(N) 118 ppm, C5 2 ppm.

Table S5: Comparison of hyperfine ^{1}H NMR shifts determined experimentally at 298 K (DIA - after subtraction the diamagnetic reference Rh($acac$)$_3$, CP - from the slope of Curie plot) and calculated (CALC) using the SO-ZORA/PBE0/TZ2P/COSMO approach with Mulliken atomic spin populations (ZORA/PBE0/TZ2P/COSMO) as implemented in AMS 2024.

Compound	Atom	δ^{HF} [ppm]			Spin Population
		DIA	CP	CALC	*10^4 [a.u.]
**EQ1**	H2	+23	+25	+46	+7
	H3	−8	−11	−13	−3
	H5	−16	−16	−25	−3
**AX1**	H2	−98	−107	−102	−17
	H3	−29	−36	−46	−6
	H5	+21	+25	+40	+6

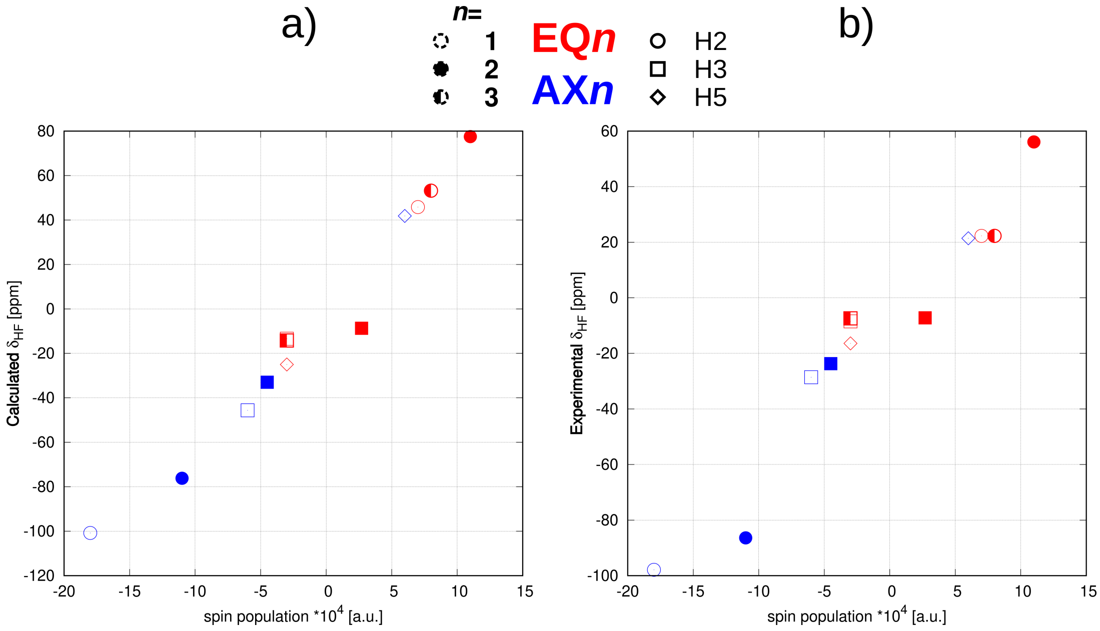

Fig. S12: Correlation of a) calculated and b) measured hyperfine ^{1}H NMR shifts with calculated spin populations of **EQ** and **AX** isomers of compounds **1-3**. For details of applied methods, see **Table S5**.

Table S6: Composition of SOMO (Löwdin % AO contributions) calculated at the UKS/PBE0/def2-TZVPP/COSMO level in *Orca 6.1* for the corresponding minimized geometry (with *acac* ring deviated from $RuCl_2O_2$ plane) and planar transition state of **EQ1**. Redistribution of spin density from π space to the plane is reflected in values of H2 A_{iso}(MHz) and hyperfine NMR shift (ppm). Note that g-tensor originates in ADF/SO-ZORA calculation.

Atom(AO)	Distorted (MIN)	Planar (TS)
Ru ($d_{x^2-y^2}$)	42.3	43.3
O1 (p_y)	3.6	3.7
O1 (p_z)	0.3	0.0
C1 (p_z)	0.2	0.0
C1 ($p_{x/y}$)	0.2	0.1
C2 (p_z)	**1.8**	0.0
C2 ($p_{x/y}$)	0.0	**0.3**
H2 (s)	0.1	0.1
A_{iso}	+1.42	+2.50
δ^{HF}	**+42.8**	**+74.9**

Because the DFT methodology used so far for the relaxation of geometry and calculation of hyperfine couplings tends to significantly overestimate the delocalization of spin density in **EQ** isomer (clearly visible on H2 and C1, **Table S3**), we tested an efficient correlation approach based on the DLPNO-CCSD methodology and calculated A_{iso}. The data summarized in **Table S7** show only marginal improvement compared to DFT. The system treated by the correlated method generally shows a lower degree of spin transfer towards the acac ligand (especially H2).

Table S7: Isotropic hyperfine coupling constants (A_{iso}) of *acac* atoms (in MHz) in **EQ1** and **AX1** from unrestricted Orca calculations: DFT, CCSD (DLPNO-HFC2/cc-pwCVTZ NoFrozenCore/COSMO), compared with the ADF results (PBE0/TZ2P/COSMO): Scalar-ZORA and SO-ZORA levels of relativity. Note origin of all geometries: ADF - ZORA/PBE0/TZ2P/COSMO.

Isomer	Atom	PBE0-ECP	CCSD	1c-ZORA	2c-ZORA
**EQ1**	H2	+1.42	+0.36	+1.26	+1.52
	H3(Me)	−0.47	−0.35	−0.64	−0.50
	C1	+3.33	+2.84	+2.33	+2.97
	C2	+4.40	+3.07	+4.77	+4.34
	C3(Me)	−0.31	−0.49	−0.29	−0.40
**AX1**	H2	−2.61	−1.94	−3.05	−3.09
	H3(Me)	−1.29	−0.84	−1.51	−1.46
	C1	−3.61	−3.28	−4.21	−4.07
	C2	+3.15	+2.29	+4.05	+3.43
	C3(Me)	−0.41	−0.51	−0.41	−0.39

Table S8: Hyperfine NMR shift (δ^{HF}) of ligand atoms (in ppm, at 298 K) in **EQ1** and **AX1** from various relativistic setups of Orca calculations (PBE0/def2-TZPP/COSMO): *ECP*, *ZORA*, and *X2C* compared with *SO-ZORA* results from ADF (SO-ZORA/PBE0/TZ2P/COSMO - corresponding ADF g-tensor was used to convert all presented A-tensors to δ^{HF}). Note Origin of all geometries: ADF - ZORA/PBE0/TZ2P/COSMO.

Isomer	Atom	*ECP*	*ZORA*	*X2C*	*SO-ZORA*	EXP
**EQ1**	H2	+43	+42	+43	+46	+23
	H3(Me)	−13	−11	−11	−13	−8
	C1	+405	+392	+400	+368	+296
	C2	+504	+511	+487	+499	*n.d.*
	C3(Me)	−34	−37	−35	−46	−31
	C4(N)-*acn*	−294	−300	−285	−320	−238
**AX1**	H2	−86	−88	−88	−102	−98
	H3(Me)	−41	−40	−40	−46	−29
	C1	−471	−475	−480	−530	−450
	C2	+418	+443	+378	+458	*n.d.*
	C3(Me)	−52	−53	−53	−50	−58
	C4(N)-*acn*	−74	+21	+6	+157	+150

References

(1) Paul, B. C.; Poddar, R. K. Synthesis, characterization and reactivity studies of dichloroacetylacetonato acetylacetone ruthenium(III). *Transition Met. Chem.* **1993**, *18*, 96 – 100.

(2) Colson, S. F.; Robinson, S. D.; Robinson, P. D.; Hinckley, C. C. Structures of *trans*- and *cis*-dichloro(1,1,1,5,5,5-hexafluoropentane-2,4-dionato-O,O')bis(triphenylphosphine)ruthenium(II). *Acta Crystallogr. Section C* **1989**, *45*, 715–718.

(3) Sheldrick, G. M. SHELXT - Integrated space-group and crystal-structure determination. *Acta Crystallographica Section A: Foundations of Crystallography* **2015**, *71*, 3 – 8.

(4) Neese, F.; Wennmohs, F.; Becker, U.; Riplinger, C. The ORCA quantum chemistry program package. *The J. Chem. Phys.* **2020**, *152*, 224108.

(5) te Velde, G.; Bickelhaupt, F. M.; Baerends, E. J.; Fonseca Guerra, C.; van Gisbergen, S. J. A.; Snijders, J. G.; Ziegler, T. Chemistry with ADF. *J. Comput. Chem.* **2001**, *22*, 931–967.

(6) Saitow, M.; Neese, F. Accurate spin-densities based on the domain-based local pair-natural orbital coupled-cluster theory. *The J. Chem. Phys.* **2018**, *149*, 034104–1 – 15.

(7) Jeremias, L.; Novotný, J.; Repisky, M.; Komorovsky, S.; Marek, R. Interplay of Through-Bond Hyperfine and Substituent Effects on the NMR Chemical Shifts in Ru(III) Complexes. *Inorg. Chem.* **2018**, *57*, 8748–8759.
